# Diverse convergent evidence in the genetic analysis of complex disease: coordinating omic, informatic, and experimental evidence to better identify and validate risk factors

**DOI:** 10.1186/1756-0381-7-10

**Published:** 2014-06-30

**Authors:** Timothy H Ciesielski, Sarah A Pendergrass, Marquitta J White, Nuri Kodaman, Rafal S Sobota, Minjun Huang, Jacquelaine Bartlett, Jing Li, Qinxin Pan, Jiang Gui, Scott B Selleck, Christopher I Amos, Marylyn D Ritchie, Jason H Moore, Scott M Williams

**Affiliations:** 1 Department of Genetics, Geisel School of Medicine at Dartmouth, Hanover, NH 03755, USA; 2 Institute for Quantitative Biomedical Sciences, Dartmouth College, Hanover, NH 03755, USA; 3 Center for Systems Genomics, Pennsylvania State University, University Park, PA 16802, USA; 4 Department of Biochemistry & Molecular Biology, Pennsylvania State University, University Park, PA 16802, USA; 5 Center for Human Genetics Research, Vanderbilt University, Nashville, TN 37232-0700, USA; 6 Community and Family Medicine, Section of Biostatistics & Epidemiology, Geisel School of Medicine, Hanover, NH 03766, USA

**Keywords:** Replication, Validation, Complex disease, Heterogeneity, GWAS, Omics, Type 2 error, Type 1 error, False negatives, False positives

## Abstract

In omic research, such as genome wide association studies, researchers seek to repeat their results in other datasets to reduce false positive findings and thus provide evidence for the existence of true associations. Unfortunately this standard validation approach cannot completely eliminate false positive conclusions, and it can also mask many true associations that might otherwise advance our understanding of pathology. These issues beg the question: How can we increase the amount of knowledge gained from high throughput genetic data? To address this challenge, we present an approach that complements standard statistical validation methods by drawing attention to both potential false negative and false positive conclusions, as well as providing broad information for directing future research. The Diverse Convergent Evidence approach (DiCE) we propose integrates information from multiple sources (omics, informatics, and laboratory experiments) to estimate the strength of the available corroborating evidence supporting a given association. This process is designed to yield an evidence metric that has utility when etiologic heterogeneity, variable risk factor frequencies, and a variety of observational data imperfections might lead to false conclusions. We provide proof of principle examples in which DiCE identified strong evidence for associations that have established biological importance, when standard validation methods alone did not provide support. If used as an adjunct to standard validation methods this approach can leverage multiple distinct data types to improve genetic risk factor discovery/validation, promote effective science communication, and guide future research directions.

## Introduction

### The validation of findings in complex disease research

The accepted gold standard for demonstrating associations in omic research settings, such as genome wide association studies, is the independent replication of preliminary findings [1]. Testing for replication involves assessing consistency by trying to repeat results in an independent sample from the original population with the same analytic approach [2]. However, many large genetic epidemiology studies and meta-analyses do not use samples from one source population, and therefore, do not attempt replication *per se*, but validation [2]. This conventional confirmation process can help to minimize false positive findings, and in doing so provides fairly compelling evidence for the existence of true associations. Although in recent years it has become evident that chance, limited power, publication bias and a variety of other factors can make this evidence less compelling than it otherwise would be [3,4]. Unfortunately, this methodology can also mask many true associations that would otherwise advance etiological research. Given that the efficacy and efficiency of research depends on reducing both false positive and false negative conclusions, validation approaches should be developed that can better prevent both types of erroneous conclusions.

If our goal is to find factors, such as genetic or environmental factors that contribute to pathophysiology, then we need to consider whether using standard validation methodology alone provides the best approach. In this paper, we propose an additional validation framework that can be used to enhance discovery and validation in omic research settings, such as transcriptome, exposome, and genome-wide association studies (GWAS).

### Shortcomings of traditional validation

Contemporary validation methods require that disease associations are observable in multiple study populations. If we acknowledge the heterogeneity of complex disease and the limitations of observational data, then we should expect that many biologically meaningful associations will not be consistently confirmed by these standard validation methods. The etiologies of complex diseases may involve multiple causal cofactors, and each of these factors may have distributions that vary greatly between study populations. We also know that observational data is often flawed; crucial variables may be unmeasured or inconsistently measured, and systematic biases can occur in ascertainment, measurement, study design, and analysis. Thus, there are numerous situations in which a true finding may fail to be confirmed using the traditional validation approach [5-7].

Additionally, current validation methods may unnecessarily inflate the rate of false negative conclusions by requiring strict multiple testing adjustments in settings where false positive conclusions could be effectively minimized with additional confirmatory data [8]. In other words, a single p-value threshold in a single analysis, no matter how strict the adjustment for multiple testing, may do a poor job of distinguishing true positive findings. Zaykin and Zhivotovsky [9] point out that the p-values for true associations tend to have ranks that are interspersed among p-values for false positive findings and that these true association p-values are often not found among the most extreme values. Thus, even strict significance thresholds cannot always separate true positive from false positive findings, and more evidence will generally be needed to determine which associations are worthy of follow-up. Multiple testing corrections can reduce type 1 errors, but they cannot solve the primary problem, that a single threshold in one analysis cannot distinguish between noise and signal of the same magnitude.

Finally, even when a finding is robust and traditional validation is observed, it still might be a false positive [7], and a consistent pattern of bias may explain the results. Careful validation protocols within one type of data should reduce false positive findings [10] but they cannot prevent false positive findings due to cryptic bias that is intrinsic to that single data type (e.g. consistent confounding in the relevant observational studies that is consistently not accounted for). In other words traditionally-validated findings that have not been examined with diverse methods may still be spurious because of systematic errors present in the single research approach used. Overall, we know that p-values have a variety of weaknesses when being used in scientific reasoning [11,12], and we should recognize these limitations by reinforcing our frameworks for discovery and validation.

### Proposed: a new approach that utilizes Diverse Convergent Evidence (DiCE)

We argue that the conventional procedures for risk factor validation could be enhanced with the addition of a supplementary method that systematically assesses diverse independent lines of evidence. This type of multifaceted strategy could provide useful information in the presence of causal heterogeneity, unrecognized bias, imperfect study designs and other settings where traditional omic validation may yield erroneous conclusions. In this approach researchers actively gather multiple distinct sources of evidence to assess a given factor (e.g., variant, gene, exposure, or pathway) in the pathophysiology of interest. Then multiple findings from various research fields can be combined to gauge whether a critical mass of evidence implicates a given factor. In this process the weaknesses of one methodology can be addressed by the complementary strengths of others; for example, evidence from knockout animal models can support information from genetic epidemiology, and findings from experimental toxicology can strengthen information from environmental epidemiology.

**Figure 1:**
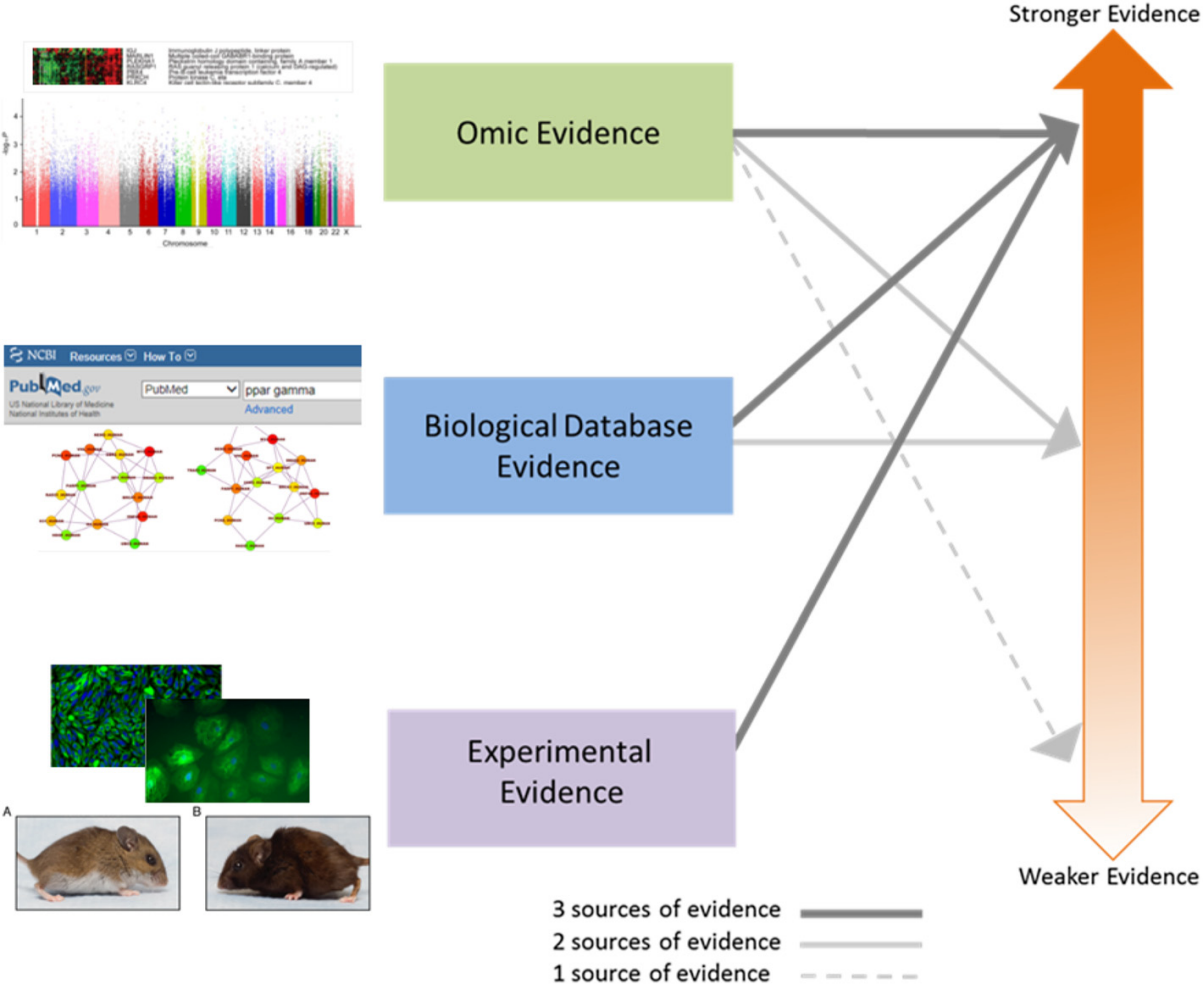
**Scoring system concept for prioritizing research findings in complex disease research.** Heat Map image adapted from [47]. Manhattan Plot image adapted from [48]. PubMed image adapted from the PubMed database website (http://www.ncbi.nlm.nih.gov/pubmed[16]) after typing in “ppar gamma” (as seen on June 9, 2014). Pathway/network image adapted from [49]. Microscopy images adapted from [50]. Mouse images adapted from [51]. Underlying images adapted from [47-51] were published under the creative commons attribution license which allows for re-use without permission (http://www.plosone.org/static/licensehttp://creativecommons.org/licenses/by/3.0/http://creativecommons.org/licenses/by/3.0/legalcode).

Here we propose a framework, Diverse Convergent Evidence (DiCE), that can help researchers to assess the importance of potential factors and decide how to proceed (Figure 1). DiCE promotes the coordination of complementary information from distinct fields to guide decisions about which findings are most worthy of follow-up efforts. When considered with the results of standard validation procedures DiCE can be used to highlight conclusions that may be erroneous (false negative or false positive) based on a systematic assessment of external knowledge. In its role as a complementary methodology DiCE does not propose a definitive endpoint or establish a single criterion for association. Rather, it distinguishes between strong and weak evidence with the intent of guiding subsequent research. This approach reflects the long-known, but rarely utilized perspective that scientific reasoning can provide guidelines but not rigid criteria for causal inference [13,14]. Typically, no single piece of evidence is necessary or sufficient for causal inference in complex disease research. If applied appropriately, the consideration of diverse lines of evidence can clarify what additional information is needed to advance our understanding of a given disease process and help investigators to apply limited resources intelligently. This framework moves beyond a single narrow approach for answering questions about complex disease to appropriately reflect etiologic and inter-dataset heterogeneity when seeking causative factors.

### Integrating evidence to calculate a DiCE score

**Figure 2:**
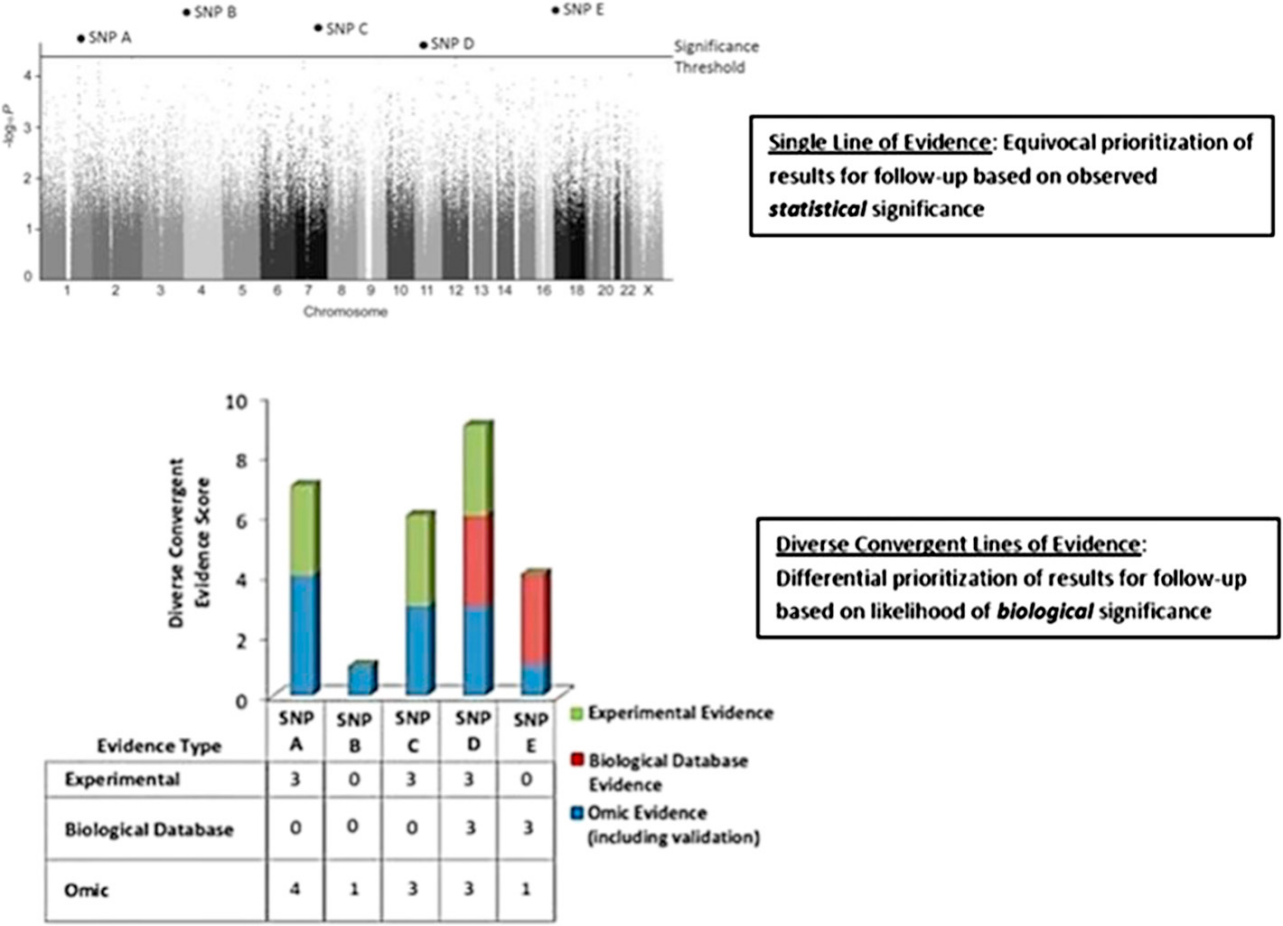
**Hypothetical DiCE scoring system implementation.** Underlying Manhattan Plot image adapted from [48]. The underlying image adapted from [48] was published under the creative commons attribution license which allows for re-use without permission (http://www.plosone.org/static/licensehttp://creativecommons.org/licenses/by/3.0/http://creativecommons.org/licenses/by/3.0/legalcode).

The DiCE system evaluates putative causal factors (e.g. genes or environmental exposures) in three broad categories of evidence: omic/observational, informatic, and laboratory experiments (Figure 1). As proposed, evidence from each category contributes to a composite score that reflects the overall strength of the evidence for a factor’s involvement in the pathophysiology of interest (Table 1 and Figure 2). The score for a given factor is elevated in the presence of diverse convergent evidence. This approach can help researchers to: 1) characterize the available evidence for a specific factor of interest; and 2) prioritize findings for further research.

**Table 1:** Diverse Convergent Evidence (DiCE) point system concept

**Omic/Observational evidence**	**Biological database (Informatic) evidence**	**Experimental (Laboratory) evidence**
Single Significant Finding	Evidence from PubMed, KEGG, GEO, GO or etc. linking the factor to the pathophysiology	Evidence from animal or cell/molecular models demonstrating a role of the factor in the pathophysiology
Yes (1 point)	Yes (3 points)	Yes (3 points)
No (0 points)	No (0 points)	No (0 points)
***And Either***		
Standard Statistical Validation (3 points) or		
Alternative Statistical Validation (2 points) or		
No Statistical Validation (0 points)		

### Omic evidence

In this framework a factor receives 1 point for being identified in an omic screening analysis (e.g. GWAS) and can then receive 3 additional points if it is validated in a second omic study using standard methods. The choice of a significance threshold is a hotly debated topic in the setting of agnostic omic scans. Because a number of significance thresholds can be defended, we leave this to the discretion of the researcher, as long as a consistent rationale is used. In our examples, we use the commonly accepted multiple testing adjusted significance threshold of p < 5 × 10^−8^[15]. To account for some of the causes of type II error in standard omic validation attempts, a risk factor that does not receive the initial 3 standard validation points can still obtain 2 points for validation through alternative exploratory approaches. Some examples of defensible alternative statistical validation attempts would include: validation by meta-analysis; validation using a distinct analytic method (e.g. PCA adjusted vs. not, using imputation vs. not etc.); or validation after accounting for a masking covariate in your analysis (e.g. stratifying or adjusting for a confounder, or considering an interacting variable). The lower point value assigned to alternative statistical validation reflects the lower quality of evidence obtained through these *post hoc* validation attempts.

### Biological database or informatic evidence

To incorporate biological database evidence, a factor can receive 3 points if supportive evidence is obtained through informatics approaches. This is a broad category that encompasses evidence obtained from biological database (pathway or network analyses) and literature searches. There are a number of approaches that could be used here, including systematic searches in PubMed [16], GEO [17], or other NCBI interfaces [18], as well as KEGG [19], GO [20], or other databases with biological annotations. Again, as with the omic evidence, the specific type of search employed here is flexible, but it should be kept consistent to generate comparable results.

### Experimental evidence

Laboratory based information is integrated into the total score by adding 3 points if there are experiments that support the involvement of this factor in the pathophysiology of interest. These experiments may include animal knockout models, cell transfections, and treatment with environmental risk factors such as nutrients, medicines, or chemicals.

Some data may fit into more than one of the three categories but a single result should only be counted once. Essentially, this process uses the sum of provisional numerical values from distinct categories of evidence to evaluate the likelihood of a given finding being true and worthy of subsequent study. We suggest that a total composite score of ≥ 6 indicates strong evidence. Although the scores themselves are arbitrary, they convey ordinal information about the available diverse evidence, and there is a strong rationale for the relationship between the component scores and the chosen threshold. No single category of evidence is necessary or sufficient to achieve a score of 6. This threshold requires convergent evidence from at least two categories, but protects the conclusion from being deleteriously affected if one category of evidence (out of the three) is missing or flawed. Overall the DiCE process yields a semi-formal dynamic heuristic that is based in logic and empiricism. The choice of search strategies for implementing the DiCE framework can vary, but a thoughtful implementation combined with an explicit description of the search details, should consistently yield useful information.

In this method the points are assigned such that roughly equal weight is given to the three categories of validation evidence (omic, informatic, and experimental). This is designed into our proposal because it is typically not appropriate to definitively pick one category *a priori* as providing better evidence. For example, information from controlled experiments may be worth more when a good and relevant disease model is available. However, there may be no appropriate assays or models available for laboratory work or the available models may not be relevant to human physiology. The situation is even worse if the research community thinks they have a pertinent disease model, but is unaware of its fundamental failings. Observational omic data that comes from humans most likely has relevance to human disease. However, issues such as measurement error and confounding may make observational data problematic, and it is not always clear when these complications are present and unaccounted for. Having approximately equal weights for the three evidence categories makes the total score relatively resilient to the known and unknown failings of each type of evidence and provides no systematic and clear bias in score assignment.

One could consider developing a more nuanced DiCE scoring rubric, by attempting to quantify the number of total validations or rate of validation successes within each evidence category. However, this approach could defeat the purpose of the method. The number of validations within one category and the validation rate within each category do not always have a clear and consistent relationship to the truth of the finding in question, and we propose that at this point they should not be folded into the rubric because of added ambiguity. These issues could be reconsidered for future modifications to the DiCE system.

Overall, DiCE is a dynamic heuristic approach that promotes the collection and integration of diverse evidence for scientific decision making. The DiCE score and the follow-up directions it suggests can change as the available evidence changes.

### Utility of the DiCE supplementary validation approach: empirical cases

#### Genetic resistance to severe malaria

In 2009 Jallow et al. published the results of a case control GWAS that searched for genetic variants associated with resistance to severe malaria [21]. This study is of interest because at the time of publication there were several previously established genetic variants that were known to confer malaria resistance, including the Hemoglobin S allele, which reduces the risk of severe malaria ten-fold. However, the Hemoglobin S signal (i.e. p-value for a marker SNP) did not achieve genome-wide significance at the 5 × 10^−8^ level [15] and none of the other known genetic risk factors (e.g., G6PD) met this criterion. The authors discussed several reasons for the failure to identify known loci, including low LD between the marker SNPs and the causal variants in their populations, and low frequencies of the causal variants in their populations. They were, however, able to attain significance by fine mapping at the Hemoglobin S locus, which was already known to associate with malaria from prior diverse evidence.

This highlights the importance of using more than one approach for causal factor identification, as information from independent lines of evidence prevented this variant from being overlooked even though it was missed by GWAS. The DiCE validation strategy proactively supports the collection of multifaceted evidence so that important signals are not missed due to the flaws of a single study, criterion, or method. Here we use the search for malaria resistance genes to demonstrate how our approach can help to characterize the strength of available evidence for specific factors and clarify future research directions.

### Hemoglobin S and malaria resistance

Using the DiCE scoring system we find that the evidence for the involvement of Hemoglobin S in malaria resistance is strong (Table 2), with a total score of 9, even though it failed traditional significance thresholds for genome wide association in the initial GWAS. The implementation details for these analyses are provided in Additional file 1. Other analytic choices could be utilized but a consistent approach should be applied throughout the implementation.

**Table 2:** Results of implementing the DiCE evidence scoring system in four contexts

**Gene and phenotype**	**Omic/Observational evidence**		**Biological database evidence**	**Experimental evidence**	Total evidence score*
	Single finding	Validation			
Hemoglobin S and malaria resistance (a positive control that would not be detected with traditional methods)	1	2	3	3	9
*ATP2B4* and malaria resistance (new lead)	1	2	3	3	9
*MARVELD3* and malaria resistance (new lead)	1	0	0	0	1
*PPARγ* and type 2 diabetes (another positive control that would not be detected with traditional methods)	0	0	3	3	6

*a total score of 6–10 is considered strong evidence.

We argue that a score of 9 provides extremely strong evidence, only possible in the presence of multiple convergent lines of evidence. In this case, we can definitively say that adherence to a single conservative analytic approach would have obscured a finding of biological interest. Rather than dismissing alternative omic analytic strategies, this system simply adjusts the score to reflect the reduced quality of statistical evidence that comes from non-traditional exploratory approaches. This example serves as a proof of principle for the DiCE approach and it also demonstrates that method can highlight reasonable directions for future research (see Additional file 1).

### *ATP2B4*, *MARVELD3*, and malaria resistance

A GWAS by Timmann et al. reported the putative association of two new genes with malaria resistance. Several SNPs were detected within the *ATP2B4* gene (encodes the primary erythrocyte calcium pump) and one SNP was identified in an intergenic region near *MARVELD3* (encodes a tight junction associated protein in vascular endothelium) [22]. Here we apply our method to these new findings to prioritize them for follow-up (see Additional file 1).

*ATP2B4* accrued an extremely strong score of 9 using our method (Table 2). Here again alternative statistical validation methods proved useful in helping to prevent an interesting lead from being overlooked. Our method also highlighted some of the next research questions related to *ATP2B4* (see Additional file 1).

The evidence summary for *MARVELD3* was much less compelling with a score of 1 (Table 2). The weakness of the evidence for *MARVELD3* at this point reflects that there is a current dearth of research on *MARVELD3* available to corroborate this finding, and this leaves open the possibility that the SNP may be a false positive finding. However in this case, a weak DiCE score also suggests another possibility: that this SNP may be linked to malaria resistance through a mechanism that does not involve *MARVELD3*. Given that the SNP is near but not in *MARVELD3*, the function of this SNP in malaria resistance (if it has one) may not involve *MARVELD3*. An exploration of other nearby genes and any known regulatory functions of this region may be fruitful in helping to identify another factor for DiCE to validate with respect to this SNP. In fact, Timmann et al. notes that the identified SNP (rs2334880) is in an intergenic region between *MARVELD3* and *TAT* (tyrosine aminotransferase) which are in a head-to-head configuration. An NCBI search [18] for “tyrosine aminotransferase and malaria” identifies a paper that implicates this enzyme in malaria pathophysiology [23] (yielding a DiCE Score of 4 for tyrosine aminotransferase). Thus perhaps it is tyrosine aminotransferase that explains the association with this SNP, and this lead may be worthy of follow-up. As always it is a judgment call, but unless more observational evidence makes *MARVELD3* more interesting, pursuing laboratory experiments for this gene is probably not warranted at this time. Importantly DiCE implementation has helped us to think systematically about the available data and it can point to next steps even when it does not point to strong conclusions.

We would argue that Timman et al. alone does not provide compelling evidence for the involvement of either *ATP2B4* or *MARVELD3* in the pathogenesis of severe malaria. Importantly, taking a single validation approach in this case does not allow these leads to be distinguished. If one only considered the p-values, these leads would be almost impossible to differentiate in terms of their relative likelihood of being etiologically relevant, because the p-values for the SNPs in *ATP2B4* (6.1 × 10^−9^, 1.5 × 10^−8^, 2.1 × 10^−8^, 5.1 × 10^−8^, 3.4 × 10^−8^) and the SNP near *MARVELD3* (3.9 × 10^−8^) are very similar. However, our simple process quickly characterized these two new leads and revealed which is currently more worthy of follow-up based on the available diverse evidence. The evidence for *ATP2B4* is strong and suggests specific new laboratory experiments, but the evidence for *MARVELD3* is weak, and therefore provides less motivation for follow-up efforts at this point. The strong performance of our method in the context of a very well established predictor of malaria resistance, Hemoglobin S, serves as a positive control (method validation), and this further suggests that the conclusions about *ATP2B4* and *MARVELD3* should be useful.

As we pointed out earlier the diverse evidence for *MARVELD3* may be weak because it has not been collected; we do not have much evidence for what we have not explicitly studied. Thus, in this case DiCE cannot provide strong evidence either way, and this is appropriate, as we would argue that strong evidence does not exist in the absence of diverse validation. However, its implementation has suggested future steps: 1) explore potential functions of this SNP that do not involve *MARVELD3* (there is evidence that tyrosine aminotransferase may explain the association between malaria and this SNP [23]), or 2) see if *MARVELD3* is detected in the next genomic screen for malaria resistance. The low DiCE score would indicate for most researchers that *MARVELD3* is not worthy of immediate laboratory follow-up. However, a researcher who already has a well characterized vascular endothelium model in their laboratory may find it worthwhile to make a *MARVELD3* knockout without additional evidence, simply because the activation energy is low for them. For other researchers, additional omic validation and some informatic evidence would likely be required to make this finding worthy of laboratory investigations.

### PPARγ and type 2 diabetes

Traditional validation has also proven to have limitations in type 2 diabetes research. In 2007 Williams et al. [24] noted that the well-established target of an entire class of type 2 diabetes drugs (PPARγ [25]) would not have been identified de novo by 3 GWA studies published that year [26-28] if traditional methods of GWAS validation were rigidly followed. The p-values for rs1801282 in the three studies were 0.019, 0.0013, and 0.0014, none of which coming close to traditional genome wide significance levels (in fact, in one of the discovery scans the index SNP had a p value of 0.83). However, the ORs were consistent (1.09 [95% CI: 1.01-1.16], 1.23 [95% CI: 1.09-1.41], 1.20 [95% CI: 1.07-1.33]), and a meta-analysis of the three studies, most likely pursued because the *PPAR*γ locus was already known based on non-GWAS-based evidence [25], yielded a small p-value, though it was still not genome wide significant (p = 1.7 × 10^−6^). In other words, an enormous amount of expensive GWAS research would not have led us to this type 2 diabetes drug target without some augmentation of traditional validation processes. However, if we apply DiCE, the method correctly characterizes the evidence for the biological relevance of *PPAR*γ as strong.

Evidence for *PPAR*γ in type 2 diabetes achieved a score of 6 (Table 2 and Additional file 1). A score of 6 is strong evidence for the involvement of *PPAR*γ, and because we already know its importance in type 2 diabetes therapy, this example serves to validate DiCE. Here we again demonstrate that adding the DiCE validation framework can allow for the detection biologically important signals where standard approaches to validation fail. The example of *PPAR*γ in type 2 diabetes also illustrates that it may be worthwhile to gather additional evidence on all hits with a p-value < 0.05 (or even p < 0.1). Furthermore, there is published evidence from the International Multiple Sclerosis Genetics Consortium which demonstrates that this type of comprehensive validation effort can be very fruitful [29]. If one is interested in filtering a large list of nominally significant findings (p < 0.05) to identify a subset most worthy of follow-up, instead of characterizing the evidence for a single finding, our flexible scoring system can be utilized in this setting as well. There will be many hits to follow-up for most complex diseases, but these efforts should be worthwhile because DiCE allows us to better interpret omic data in light of other biologically relevant signals.

## Discussion

In this paper we have proposed a supplemental analytic framework (DiCE) to improve discovery and validation performance in omic research settings such as GWAS. This method promotes the collection of diverse evidence in order to leverage its inherent resistance to the systematic failings that are possible with single approaches. Additionally it allows for the coordination of varied evidence to effectively guide future research. We have also illustrated the validity and utility of the DiCE strategy using four case studies: two proof of principle examples and two exploratory examples. The proposed scoring system is subjective, as is a nominal p of 0.05, but it accomplishes the major goal of combining multiple data types into a unified framework for evidence assessment.

R.A. Fisher, the father of p-value based inference, provides us with evidence that the application of a thoughtful yet subjective convention can be very productive. He did not view the 5 % false positive rate threshold as an immutable postulate but rather as a convenient evidence benchmark that could guide scientific decision making [30,31]. “*If P is between 0.1 and 0.9 there is certainly no reason to suspect the hypothesis tested. If it is below 0.02 it is strongly indicated that the hypothesis fails to account for the whole of the facts. We shall not often be astray if we draw a conventional line at 0.05 . . .*” [32] Thus, much of our biomedical research progress in the last 80 years has been based on a metric that is subjective and imperfect, but useful. We propose that we can address some of these imperfections and better identify important biological results by considering additional carefully chosen guidelines.

The overall objective of DiCE is to encourage the collection of data in several categories, since no single category is typically necessary or sufficient to supply compelling evidence of causation. With this method if one category of evidence is unavailable or biased the direction of future research will not necessarily be deleteriously altered. In addition, this approach depends on interdisciplinary coordination, which can build bridges among researchers from disparate fields, improving the speed and quality of discovery.

Of course, as with any approach to evidence synthesis the efficacy of this method will depend on the quality of the available prior studies and their annotation as well as the technology used to access this information. The utility of this strategy will be limited where relevant information does not exist, is derived from flawed studies, or is difficult to access. Researchers with expertise in the relevant subject matter and methodologies should be consulted when the value of a piece of evidence is in question. Furthermore, Chanock et al. 2007 provides a detailed list of considerations to help guide researchers when making study quality assessments [1]. These judgments may be particularly important in the context of low quality omic studies that could provide a poor foundation for directing further inquiry. Essentially, this approach will be useful where it is thoughtfully applied. Furthermore, with the advent of modern text-mining methods this approach can be semi-automated for use in high throughput examination of multiple findings prior to human interpretation.

Widespread application of DiCE also has the potential to increase the credibility of biomedical research by appropriately conveying uncertainty to all audiences and increasing likelihood that highly publicized findings will have biological relevance. Reviewers and editors may still require a specific level of statistical evidence (e.g. p < 5 × 10^−8^), but with the addition of a DiCE score both significant and non-significant p-values can be better contextualized in terms of their likelihood of having biological relevance in the pathophysiology of interest. Published findings will be as accessible as they were before DiCE, but bold interpretation, publicity, and translation attempts will be hard to defend in the context of a low DiCE score. A DiCE score can allow readers to quickly gauge the corroborating evidence from beyond the paper they are reading, and a low DiCE score can encourage the lay press to include appropriate caveats in their reports or to wait until the evidence is stronger before reporting. If a preliminary finding is exciting and diverse evidence has not been collected, a low DiCE score should encourage researchers to collect the remaining evidence without delay, and thus the quality of the finding should be quickly ascertained. Thus DiCE scores can be expected to have a dynamic and productive interplay with the literature. Overall, this method should improve the research dissemination process by providing a simple metric for journals, researchers, the media, and the general public to better vet findings. Further, by providing a diverse range of evidence, a wider range of domain experts can weigh in on scientific findings, rather than with the current scenario where most results are reported to and evaluated by a very specific group of domain experts. This should promote the broad evaluation and sharing of a given set of results, allowing for better guidance and coordination future research directions.

### Logistics: DiCE scores can be quickly added and easily incorporated into any GWAS report

DiCE is designed to provide information that complements standard statistical validation methods. Thus DiCE can be used to systematically characterize GWAS significant hits to assess for the likelihood of false positive conclusions and suggest future research directions. It can also be used to characterize a small number of sub-threshold statistical associations (e.g. those with the 10 smallest sub-threshold p-values) to assess for the likelihood of false negative conclusions. The utility of DiCE may be expanded with the development of semi-automated procedures for calculating DiCE scores. With semi-automated implementation protocols DiCE could be applied to all nominally significant GWAS findings to detect possible false negative conclusions in this larger group.

### Why allow for omic analytic strategies that do not adhere to rigid multiple testing adjustments?

Strict multiple testing correction results in the inefficient use of expensive data. Omics technologies such as GWAS can produce a list of candidate factors enriched for answers, but they cannot produce a list of answers. Our traditional omic analytic methods for the discovery of factors influencing pathology implicitly assume that complex diseases have simple etiologies (i.e. no covariates or interactions will affect independent validation), and that observational data is virtually devoid of cryptic bias, confounding, and measurement error. If we strictly adhere to simplistic models, we will fail to access the substantial amount of knowledge that is embedded in findings that fail standard validation.

The desire to require extremely small p-values flows, in part, from the laudable aspiration to reduce the number of false positive findings. However, this approach increases the likelihood of false negative conclusions, the cost of which is not trivial. The requirement of very small p-values also reflects the expectation that simple answers will flow from omic tools. We use significance thresholds that give us a small number of answers to consider, and squeeze datasets so tightly that only the most extreme findings are considered valid. Accepted omic results may only be this extreme from a combination of true effects and chance. To obtain a GWAS significant result, one needs: 1) a large effect size; 2) a precise effect estimate; or 3) luck. Large effect sizes are uncommon in complex disease; therefore, we attempt to increase the precision of estimates with large sample sizes (which may be counterproductive if heterogeneity is increased when adding participants). However, we often depend on the luck of the draw (cf. “winners curse”) [33,34], when we insist on extreme levels of certainty from a single analysis.

It should also be noted here that the rationale behind multiple testing adjustments and their use in certain contexts has been exposed to important criticisms in the last 25 years [35]. In 1990 Kenneth Rothman proposed that attempting to reduce the number of false positive findings with multiple testing adjustments can hinder observation and impede the advancement of science. *“An association that would have been interesting to explore if examined alone can thus be converted to one that is worth much less attention if judged by the criteria based on [multiple comparison] adjustments. Since other associations in the set of comparisons may have no bearing on the one in question, the upshot is that irrelevant information from the data can diminish the informativeness of an association of possible interest.”*

With the advent of omic research designs, and the development of new options for multiple testing adjustment, Rothmans’s analysis has become even more important, and a number of authors have extended his comments, including Bender and Lange [36]: *“ . . . in exploratory studies without prespecified hypotheses there is typically no clear structure in the multiple tests, so an appropriate multiple test adjustment is difficult or even impossible. Hence we prefer that data of exploratory studies are analyzed without multiplicity adjustment . . . To confirm these results, the corresponding hypotheses have to be tested in confirmatory studies.”*

Recently, Williams and Haines revisited and extended these lines of thought [8]. They emphasized that relative importance of type I and type II error is dependent on the stage of the research, and that requiring both multiple testing correction and independent validation causes an unacceptable number of meaningful leads to be ignored. If one is early in the discovery process and has the capacity to gather follow-up evidence then type II errors should be of greater concern because the type I errors will be corrected but the type II errors will not be. *“We argue that when examining an array of nominally positive findings, statistical stringency alone does not permit us to determine which findings are by chance and which are not, and therefore, setting too stringent cutoff for Type I error criterion for association decreases power to find real associations.”*

DiCE further extends these ideas by emphasizing that the processes of validation should be dependent on diverse evidence, because this better addresses the type I/type II error problem, as well as other recognized and unrecognized weaknesses of omic analyses. With this method we have not abandoned our concern for type I error. We still value the evidence that comes from small p-values and rely on independent confirmation, but we now deliberately acknowledge the importance of type II errors and proactively attempt to reduce them.

To the extent that our approach streamlines the consideration of diverse convergent evidence, it can speed up the progression from omic findings to interventions.

### Comparison of DiCE to existing procedures for knowledge integration

DiCE is a semiformal, dynamic heuristic that reflects the strength of available diverse convergent evidence, and it is designed to supplement standard statistical validation procedures. This makes it different from most statistical analytic approaches, but there are some useful comparisons to be made with other methods. In particular, discussing Meta-analysis, Inter-Rater Reliability, and Inference Ranking techniques should help to contextualize the role of DiCE in high throughput genetic research.

In genetic research meta-analysis typically utilizes fixed effect models to integrate information from multiple observational studies to estimate a single association magnitude (and p-value) for a given SNP [37]. Thus, it is most useful when the association magnitude for a given variable is effectively universal (i.e. not context dependent). In contrast, DiCE scores integrate available information from observational studies, biological databases, and experiments to provide a structured assessment of the likelihood of biological relevance of a given SNP. This is very useful information when the observed association magnitudes vary by context (genetic or environmental background), or the observational studies share a consistent bias that generates a precise yet inaccurate association magnitude.

The fixed effect meta-analyses typically used in discovery genomics settings obtain a single omnibus effect estimate by assuming the heterogeneity of effect size is due to random error [37]. However, we know that context dependent associations and differential biases can also generate heterogeneity. Thus, this assumption may often be unreasonable, and there is a need for discovery approaches that have utility when the effect size variation is not due to random error. Random effect meta-analyses can better account for population specific heterogeneity but they still yield one “average” association magnitude, and they likely won’t clarify if the heterogeneity is due to context dependent effect sizes or differential biases. However, DiCE should have utility in these settings because the DiCE score is unlikely to be elevated when significant omic findings are driven purely by bias or random error. A high DiCE score in the context of heterogeneous omic study results suggests that the significant associations may be due to a true context dependent association rather than random error or differential bias. A low DiCE score in this setting suggests that random error or differential bias may have generated the significant associations, or that relevant informatic/experimental evidence has not yet been collected.

Conceptually the DiCE score is similar to an ordinal inter-rater reliability (IRR) metric [38] in a setting where there are 3 “raters”: omics, informatics, and experiments. Each “rater” provides an assessment: found any evidence of biological relevance or found no evidence of biological relevance. More concordant responses result in a higher DiCE score. Important comparisons can also be made with inference ranking systems from Environmental Health and high-throughput Toxicology. Because it is not currently feasible for researchers to thoroughly assess the safety of every chemical that humans may be exposed to, researchers must prioritize their efforts to identify chemicals likely to pose the greatest risk to public health based on currently available evidence. In this setting, integrating diverse evidence into a rank score helps guide the direction of future research as well as facilitate science communication and decision making [39,40]. Thus, diverse evidence based prioritization systems have established their utility in an analogous high throughput data setting. Furthermore, approaches that leverage convergent evidence have already shown some utility in guiding genetic analyses [41-44], and now DiCE expands this concept and provides an accessible protocol that should facilitate its wider use.

### Conclusion: Diversify validation strategies to advance the progress of research

In this paper we present a new method (DiCE) for improving the detection and validation of relevant biological signals in omic data by proactively considering diverse evidence. This approach provides a chance to strengthen our validation strategies and advance the progress of research. We argue that DiCE, when properly implemented, should leverage multidisciplinary information to reduce rates of both false positive and false negative conclusions. Standard validation protocols implicitly assume that there is one truth (i.e. a marginal finding) and it will be discoverable no matter what the contextual background (covariates, biases, confounding). Furthermore, these validation procedures, when used in isolation, can lead to incorrect conclusions when there is a consistent bias in the observational studies. Therefore, many causal factors will go unnoticed and some meaningless “hits” may be over-interpreted without the development of additional validation approaches, such as DiCE.

The utility of gathering diverse classes of evidence in the context of complex disease research is not a new idea [45], but in current research practice the simplicity and allure of rigid statistical criteria often overshadows this basic concept. We should not forget that statistical criteria are very important tools but not substitutes for more complete scientific investigation and reasoning. Our framework is designed to promote this kind of comprehensive scientific reasoning. The recent improvements in observational research technologies/algorithms, informatics/systems biology resources, and laboratory based disease models have the potential to greatly advance research efficiency and productivity, if thoughtfully coordinated. These guidelines should promote the synergy that will allow these technologies to deliver on their promises.

### Definition of terms as used here

#### Replication

An attempt to assess the consistency of association by trying to repeat the results in an independent sample from the original population with the same analytic approach [2].

### Validation

An attempt to assess the consistency and generalizability of association by trying to repeat the results in an independent sample from a different population using either the same analytic method or a different approach [2].

### Cofactor

A component cause or causal cofactor (e.g. biological factors that physically interact to generate pathogenic mechanisms). Component causes are factors that are insufficient to cause disease by themselves but can help cause disease when they occur with other component causes. For more details see [46].

### Covariate

A variable that may impact the estimated association between the variable of primary interest and the outcome (via confounding, interaction, and etc.) A covariate may have this impact through causal or non-causal (correlational) relationships. If not properly considered in the analysis covariates may generate bias in the estimated association between the variable of primary interest and the outcome. Cofactors are covariates that may influence estimated associations through causal mechanisms.

## Abbreviations

GWAS: Genome-wide association studies.

## Competing interests

The authors declare that they have no competing interest.

## Authors’ contributions

This paper evolved out of discussions at the EDGE 2013 conference organized by MDR and JHM. TC drafted the manuscript and proposed the scoring methodology which developed as a product of exchanges with the co-authors. SP and MW drafted the figures. NK and SMW provided editorial and intellectual content review. All co-authors provided important input or feedback. All authors read and approved the final manuscript.

## Supplementary Material

Additional file 1 Implementation examples for the Diverse Convergent Evidence (DiCE) Scoring System. Click here for file
