# Supplementary material for: Diverse convergent evidence in the genetic analysis of complex disease: coordinating omic, informatic, and experimental evidence to better identify and validate risk factors
Source: BioData Min. 2014 Jun 30;7:10. doi: 10.1186/1756-0381-7-10 (PMC4112852; doi:10.1186/1756-0381-7-10)
Supplement: Additional file 1 — Implementation examples for the Diverse Convergent Evidence (DiCE) Scoring System. [file 1756-0381-7-10-S1.docx]

**Additional file 1:**

**Implementation examples for the Diverse Convergent Evidence (DiCE) Scoring System**

**Hemoglobin S and Malaria Resistance**

If we use the DiCE scoring system approach we find that the evidence for the involvement of Hemoglobin S in Malaria Resistance is strong even though it initially failed traditional significance thresholds for genome wide association. There are many ways to properly use our framework and what follows is a description of one appropriate implementation.

Single omic/observational finding: 1 point. Timmann et al. found a genome wide significant association at p < 5 x 10^-8^ [[1](file:///C:\Users\Tim\Documents\dartmouth\EDGE%20conference\stuff%20for%20submission\files%20for%20submission\Supplemental%20Materials%20for%20Diverse%20Convergent%20Evidence%20Manuscript.docx#_ENREF_1)], between a marker SNP for the hemoglobin beta locus (hemoglobinS is a variant at this locus) and resistance to severe malaria. [[2](file:///C:\Users\Tim\Documents\dartmouth\EDGE%20conference\stuff%20for%20submission\files%20for%20submission\Supplemental%20Materials%20for%20Diverse%20Convergent%20Evidence%20Manuscript.docx#_ENREF_2)]

Alternative statistical validation using a distinct method: 2 points. Jallow et al. failed to identify a genome wide significant association with their marker SNP for the hemoglobin beta locus, but after imputation, the signal for the hemoglobin S SNP (in the hemoglobin beta locus) was significant. [[3](file:///C:\Users\Tim\Documents\dartmouth\EDGE%20conference\stuff%20for%20submission\files%20for%20submission\Supplemental%20Materials%20for%20Diverse%20Convergent%20Evidence%20Manuscript.docx#_ENREF_3)]

Biological database or informatics based evidence: 3 points. A simple search at the NCBI website (https://www.ncbi.nlm.nih.gov, accessed 5-7-13) with the following query: “hemoglobinS and malaria resistance” yielded 139 papers in PubMed and 5 OMIM entries. The first PubMed entry listed is a review of many studies linking hemoglobin S to malaria resistance. [[4](file:///C:\Users\Tim\Documents\dartmouth\EDGE%20conference\stuff%20for%20submission\files%20for%20submission\Supplemental%20Materials%20for%20Diverse%20Convergent%20Evidence%20Manuscript.docx#_ENREF_4)] A search for “hemoglobinS and malaria” (https://www.ncbi.nlm.nih.gov, accessed 3-4-14) returned a large group of 1201 papers in PubMed that also includes this paper. Note that we have chosen to use the NCBI site for our biological database approach in these examples because it is comprehensive and accessible, but a variety of alternative informatics tools could have been used here.

Experimental evidence: 3 points. Looking further into the literature we find experiments have demonstrated several specific physiologic mechanisms that could explain why hemoglobin S carriers have increased resistance to severe malaria. [[5-8](file:///C:\Users\Tim\Documents\dartmouth\EDGE%20conference\stuff%20for%20submission\files%20for%20submission\Supplemental%20Materials%20for%20Diverse%20Convergent%20Evidence%20Manuscript.docx#_ENREF_5)] Parasitized erythrocytes from hemoglobin S carriers have reduced PfEMP1 (a parasite cytoadherance ligand/virulence factor) on their surface and demonstrate reduced binding to microvascular endothelial cells. [[5](file:///C:\Users\Tim\Documents\dartmouth\EDGE%20conference\stuff%20for%20submission\files%20for%20submission\Supplemental%20Materials%20for%20Diverse%20Convergent%20Evidence%20Manuscript.docx#_ENREF_5)] This may reduce the vascular complications of severe malaria. Parasitized erythrocytes from hemoglobin S carriers are also more likely to sickle than non-parasitized erythrocytes, which may make them more likely to be removed from circulation. [[6](file:///C:\Users\Tim\Documents\dartmouth\EDGE%20conference\stuff%20for%20submission\files%20for%20submission\Supplemental%20Materials%20for%20Diverse%20Convergent%20Evidence%20Manuscript.docx#_ENREF_6)] Pasvol et al. reported decreased invasion and growth of parasites in erythrocytes of hemoglobin S carriers in low oxygen conditions; this effect did not appear to depend on sickling. [[7](file:///C:\Users\Tim\Documents\dartmouth\EDGE%20conference\stuff%20for%20submission\files%20for%20submission\Supplemental%20Materials%20for%20Diverse%20Convergent%20Evidence%20Manuscript.docx#_ENREF_7)] Furthermore, Ayi et al. showed that erythrocytes of hemoglobin S carriers in an early (ring) stage of infection are more likely to be phagocytized by monocytes. [[8](file:///C:\Users\Tim\Documents\dartmouth\EDGE%20conference\stuff%20for%20submission\files%20for%20submission\Supplemental%20Materials%20for%20Diverse%20Convergent%20Evidence%20Manuscript.docx#_ENREF_8)]

Evidence summary for the involvement of hemoglobin S in malaria resistance: 1 + 2 + 3 + 3 = 9.

***ATP2B4* and Malaria Resistance**

Here we use DiCE to summarize the evidence for the involvement of *ATP2B4* (encodes the primary erythrocyte calcium pump), in malaria resistance [[2](file:///C:\Users\Tim\Documents\dartmouth\EDGE%20conference\stuff%20for%20submission\files%20for%20submission\Supplemental%20Materials%20for%20Diverse%20Convergent%20Evidence%20Manuscript.docx#_ENREF_2)], and it is not clear *a priori* what the strength of the convergent evidence will be.

Single omic/observational finding: 1 point. Timmann et al. found genome wide significant associations between SNPs in *ATP2B4* and resistance to severe malaria. [[2](file:///C:\Users\Tim\Documents\dartmouth\EDGE%20conference\stuff%20for%20submission\files%20for%20submission\Supplemental%20Materials%20for%20Diverse%20Convergent%20Evidence%20Manuscript.docx#_ENREF_2)]

Alternative statistical validation by meta-analysis: 2 points. Timmann et al. then went back to the Jallow et al. data and failed to identify a genome wide significant associations with these SNPs, but the ORs were of very similar magnitude, the p-values were all <0.05, and the SNPs were genome wide significant by meta-analysis. [[2](file:///C:\Users\Tim\Documents\dartmouth\EDGE%20conference\stuff%20for%20submission\files%20for%20submission\Supplemental%20Materials%20for%20Diverse%20Convergent%20Evidence%20Manuscript.docx#_ENREF_2), [3](file:///C:\Users\Tim\Documents\dartmouth\EDGE%20conference\stuff%20for%20submission\files%20for%20submission\Supplemental%20Materials%20for%20Diverse%20Convergent%20Evidence%20Manuscript.docx#_ENREF_3)]

Biological database or informatics based evidence: 3 points. A simple search at the NCBI website (https://www.ncbi.nlm.nih.gov, accessed 5-9-13) with the following query: “ATP2B4 and malaria” yielded 2 papers in PubMed and 2 OMIM entries. One of the PubMed entries was Timmann et al., but the other was a new candidate gene study which reported significant associations between an *ATP2B4* SNP and the prevalence of peripheral/placental malaria in primiparous women. [[9](file:///C:\Users\Tim\Documents\dartmouth\EDGE%20conference\stuff%20for%20submission\files%20for%20submission\Supplemental%20Materials%20for%20Diverse%20Convergent%20Evidence%20Manuscript.docx#_ENREF_9)]

Experimental evidence: 3 points. Laboratory experiments have shown that lowering the calcium level in the parisitophorous vacuole of the erythrocyte (the vacuole the parasite lives in) impedes the maturation of malaria parasites. [[2](file:///C:\Users\Tim\Documents\dartmouth\EDGE%20conference\stuff%20for%20submission\files%20for%20submission\Supplemental%20Materials%20for%20Diverse%20Convergent%20Evidence%20Manuscript.docx#_ENREF_2), [10](file:///C:\Users\Tim\Documents\dartmouth\EDGE%20conference\stuff%20for%20submission\files%20for%20submission\Supplemental%20Materials%20for%20Diverse%20Convergent%20Evidence%20Manuscript.docx#_ENREF_10)] Platelet and vascular endothelial cells are also involved in malaria pathophysiology [[2](file:///C:\Users\Tim\Documents\dartmouth\EDGE%20conference\stuff%20for%20submission\files%20for%20submission\Supplemental%20Materials%20for%20Diverse%20Convergent%20Evidence%20Manuscript.docx#_ENREF_2), [11](file:///C:\Users\Tim\Documents\dartmouth\EDGE%20conference\stuff%20for%20submission\files%20for%20submission\Supplemental%20Materials%20for%20Diverse%20Convergent%20Evidence%20Manuscript.docx#_ENREF_11), [12](file:///C:\Users\Tim\Documents\dartmouth\EDGE%20conference\stuff%20for%20submission\files%20for%20submission\Supplemental%20Materials%20for%20Diverse%20Convergent%20Evidence%20Manuscript.docx#_ENREF_12)] and they both are activated by intracellular calcium. [[2](file:///C:\Users\Tim\Documents\dartmouth\EDGE%20conference\stuff%20for%20submission\files%20for%20submission\Supplemental%20Materials%20for%20Diverse%20Convergent%20Evidence%20Manuscript.docx#_ENREF_2), [13](file:///C:\Users\Tim\Documents\dartmouth\EDGE%20conference\stuff%20for%20submission\files%20for%20submission\Supplemental%20Materials%20for%20Diverse%20Convergent%20Evidence%20Manuscript.docx#_ENREF_13), [14](file:///C:\Users\Tim\Documents\dartmouth\EDGE%20conference\stuff%20for%20submission\files%20for%20submission\Supplemental%20Materials%20for%20Diverse%20Convergent%20Evidence%20Manuscript.docx#_ENREF_14)]

Evidence summary for the involvement of ATP2B4 in malaria resistance: 1 + 2 + 3 + 3 = 9.

***MARVELD3* and Malaria Resistance**

Here we use our framework to summarize the evidence for its involvement *MARVELD3*, (encodes a tight junction associated protein in vascular endothelium), in malaria resistance [[2](file:///C:\Users\Tim\Documents\dartmouth\EDGE%20conference\stuff%20for%20submission\files%20for%20submission\Supplemental%20Materials%20for%20Diverse%20Convergent%20Evidence%20Manuscript.docx#_ENREF_2)], and it is not clear *a priori* what the strength of the convergent evidence will be.

Single omic/observational finding: 1 point. Timmann et al. found a genome wide significant association between an imputed intergenic SNP 6.4 kb upstream of *MARVELD3* and resistance to severe malaria. [[2](file:///C:\Users\Tim\Documents\dartmouth\EDGE%20conference\stuff%20for%20submission\files%20for%20submission\Supplemental%20Materials%20for%20Diverse%20Convergent%20Evidence%20Manuscript.docx#_ENREF_2)]

No statistical validation, traditional or alternative: 0 points. Timmann et al. went back to the Jallow et al. data and failed to identify a genome wide significant association, in fact the p value was 0.6, and the ORs were not consistent (1.24 [95%CI: 1.15-1.34] vs. 0.96 [0.81-1.13]). [[2](file:///C:\Users\Tim\Documents\dartmouth\EDGE%20conference\stuff%20for%20submission\files%20for%20submission\Supplemental%20Materials%20for%20Diverse%20Convergent%20Evidence%20Manuscript.docx#_ENREF_2), [3](file:///C:\Users\Tim\Documents\dartmouth\EDGE%20conference\stuff%20for%20submission\files%20for%20submission\Supplemental%20Materials%20for%20Diverse%20Convergent%20Evidence%20Manuscript.docx#_ENREF_3)]

Biological database or informatics based evidence: 0 points. A simple search at the NCBI website (https://www.ncbi.nlm.nih.gov, accessed 5-10-13) with the following query: “MARVELD3 and malaria” yielded 1 paper in Pubmed and 2 OMIM entries. The Pubmed entry was the Timmann et al. paper and the OMIM entries were based on the Timmman et al. findings along with general description of *MARVELD3*.

Experimental evidence: 0 points. Timmann et al. speculate that tight junctions in vascular endothelium could be involved in severe malaria vasculopathy [[2](file:///C:\Users\Tim\Documents\dartmouth\EDGE%20conference\stuff%20for%20submission\files%20for%20submission\Supplemental%20Materials%20for%20Diverse%20Convergent%20Evidence%20Manuscript.docx#_ENREF_2)] but there does not appear to be direct laboratory evidence for this.

Evidence summary for the involvement of *MARVELD3* in malaria resistance: 1 + 0 + 0 + 0 = 1.

Note that upon meta-analysis Timmann et al. reported a p-value of 1.9x10^-6^ for *MARVELD3*. This does not meet our stated significance threshold of p < 5 x 10^-8^. [[1](file:///C:\Users\Tim\Documents\dartmouth\EDGE%20conference\stuff%20for%20submission\files%20for%20submission\Supplemental%20Materials%20for%20Diverse%20Convergent%20Evidence%20Manuscript.docx#_ENREF_1)] However, even if we were to consider the meta-analysis as 1 test (p<0.05 threshold), with only two studies involved, it is evident that the p-value for this omnibus association is completely driven by the Timmann et al data. We would not consider this good meta-analytic evidence even using a p<0.05 threshold. If you did consider this good evidence, then the total score for *MARVELD3* would become a 3 and the overall conclusion would be qualitatively the same.

***PPARγ* and Type 2 Diabetes**

Finally we use DiCE to consider the involvement of *PPARγ* in Type 2 diabetes. This will serve as another positive control (method validation) because the role of *PPARγ* in this biology is already well established. [[15](file:///C:\Users\Tim\Documents\dartmouth\EDGE%20conference\stuff%20for%20submission\files%20for%20submission\Supplemental%20Materials%20for%20Diverse%20Convergent%20Evidence%20Manuscript.docx#_ENREF_15), [16](file:///C:\Users\Tim\Documents\dartmouth\EDGE%20conference\stuff%20for%20submission\files%20for%20submission\Supplemental%20Materials%20for%20Diverse%20Convergent%20Evidence%20Manuscript.docx#_ENREF_16)]

No single omic/observational finding: 0 points. In 2007 Williams et al. [[16](file:///C:\Users\Tim\Documents\dartmouth\EDGE%20conference\stuff%20for%20submission\files%20for%20submission\Supplemental%20Materials%20for%20Diverse%20Convergent%20Evidence%20Manuscript.docx#_ENREF_16)] noted that the target of an entire class of type 2 diabetes drugs (*PPARγ*  [[15](file:///C:\Users\Tim\Documents\dartmouth\EDGE%20conference\stuff%20for%20submission\files%20for%20submission\Supplemental%20Materials%20for%20Diverse%20Convergent%20Evidence%20Manuscript.docx#_ENREF_15)]) would not have been identified *de novo* by 3 GWA studies published that year [[17-19](file:///C:\Users\Tim\Documents\dartmouth\EDGE%20conference\stuff%20for%20submission\files%20for%20submission\Supplemental%20Materials%20for%20Diverse%20Convergent%20Evidence%20Manuscript.docx#_ENREF_17)] if traditional methods of GWAS validation were rigidly followed. The p-values for rs1801282 of 0.019, 0.0013, and 0.0014 did not come close to traditional genome wide significance levels. However, the ORs were consistent (1.09 [95%CI: 1.01-1.16], 1.23 [95%CI: 1.09-1.41], 1.20 [95%CI: 1.07-1.33]), and a meta-analysis of the three studies, which was most likely done because the *PPARγ* locus was already known based on non-GWAS-based evidence [[15](file:///C:\Users\Tim\Documents\dartmouth\EDGE%20conference\stuff%20for%20submission\files%20for%20submission\Supplemental%20Materials%20for%20Diverse%20Convergent%20Evidence%20Manuscript.docx#_ENREF_15)], resulted in a small p-value (p = 1.7 x 10^-6^). Nevertheless, as Williams et al. point out these 3 studies did not provide a genome-wide significant omic finding.

No statistical validation: 0 points. These studies did provide meta-analytic evidence for an association, but as we are using a traditional p < 5 x 10^-8^ [[1](file:///C:\Users\Tim\Documents\dartmouth\EDGE%20conference\stuff%20for%20submission\files%20for%20submission\Supplemental%20Materials%20for%20Diverse%20Convergent%20Evidence%20Manuscript.docx#_ENREF_1)] significance threshold for these examples, we will not consider this data to provide alternative statistical validation. [[17-19](file:///C:\Users\Tim\Documents\dartmouth\EDGE%20conference\stuff%20for%20submission\files%20for%20submission\Supplemental%20Materials%20for%20Diverse%20Convergent%20Evidence%20Manuscript.docx#_ENREF_17)]

Biological database or informatics based evidence: 3 points. A simple search at the NCBI website (https://www.ncbi.nlm.nih.gov, accessed 5-21-13) with the following query: “PPARG and type 2 diabetes” yielded 1429 papers in PubMed and 27 OMIM entries.

Experimental evidence: 3 points. In terms of laboratory evidence, it has been known for years that certain anti-diabetic drugs (Thiazolidinediones) bind PPARγ [[15](file:///C:\Users\Tim\Documents\dartmouth\EDGE%20conference\stuff%20for%20submission\files%20for%20submission\Supplemental%20Materials%20for%20Diverse%20Convergent%20Evidence%20Manuscript.docx#_ENREF_15)], and now there is evidence that blocking CDK5-mediated phosphorylation of PPARγ may result in anti-diabetic effects with fewer side effects than PPARγ agonism. [[20](file:///C:\Users\Tim\Documents\dartmouth\EDGE%20conference\stuff%20for%20submission\files%20for%20submission\Supplemental%20Materials%20for%20Diverse%20Convergent%20Evidence%20Manuscript.docx#_ENREF_20)] A recent review by Rosenson et al discusses how advances in our understanding of PPARγ physiology may lead to safer small molecules for type 2 diabetes treatment. [[21](file:///C:\Users\Tim\Documents\dartmouth\EDGE%20conference\stuff%20for%20submission\files%20for%20submission\Supplemental%20Materials%20for%20Diverse%20Convergent%20Evidence%20Manuscript.docx#_ENREF_21)]

Evidence summary for the involvement of PPAR-gamma in type 2 diabetes: 0 + 0 + 3 + 3 = 6.

Note: If we had decided to consider the meta-analytic approach to *PPARγ* as a single test then we could appropriately use p<0.05 as our significance threshold, and thus p = 1.7 x 10^-6^ would meet this criterion. This would yield a DiCE score of 8, and in this case, the qualitative *PPARγ* conclusion would not change, because of the presence of substantial non-omic evidence. Though not as impressive as a score of 8, a score of 6 still represents strong convergent evidence. Again this situation argues against the indiscriminant use of extreme significance thresholds in omic scans, and argues for following up on all hits at p<0.05. If the other categories of evidence had not existed, *PPARγ* would not have been identified as an interesting lead in the omic scans. However, the use of a nominal cut-off (p<0.05) would likely require semi-automated means to assess alternative data types for the many hits.

**References**

1. Dudbridge F, Gusnanto A: **Estimation of significance thresholds for genomewide association scans.** *Genetic epidemiology* 2008, **32:**227-234.

2. Timmann C, Thye T, Vens M, Evans J, May J, Ehmen C, Sievertsen J, Muntau B, Ruge G, Loag W, Ansong D, Antwi S, Asafo-Adjei E, Nguah SB, Kwakye KO, Akoto AO, Sylverken J, Brendel M, Schuldt K, Loley C, Franke A, Meyer CG, Agbenyega T, Ziegler A, Horstmann RD: **Genome-wide association study indicates two novel resistance loci for severe malaria.** *Nature* 2012, **489:**443-446.

3. Jallow M, Teo YY, Small KS, Rockett KA, Deloukas P, Clark TG, Kivinen K, Bojang KA, Conway DJ, Pinder M, Sirugo G, Sisay-Joof F, Usen S, Auburn S, Bumpstead SJ, Campino S, Coffey A, Dunham A, Fry AE, Green A, Gwilliam R, Hunt SE, Inouye M, Jeffreys AE, Mendy A, Palotie A, Potter S, Ragoussis J, Rogers J, Rowlands K*, et al*: **Genome-wide and fine-resolution association analysis of malaria in West Africa.** *Nature genetics* 2009, **41:**657-665.

4. Bunn HF: **The triumph of good over evil: protection by the sickle gene against malaria.** *Blood* 2013, **121:**20-25.

5. Cholera R, Brittain NJ, Gillrie MR, Lopera-Mesa TM, Diakite SA, Arie T, Krause MA, Guindo A, Tubman A, Fujioka H, Diallo DA, Doumbo OK, Ho M, Wellems TE, Fairhurst RM: **Impaired cytoadherence of Plasmodium falciparum-infected erythrocytes containing sickle hemoglobin.** *Proceedings of the National Academy of Sciences of the United States of America* 2008, **105:**991-996.

6. Luzzatto L, Nwachuku-Jarrett ES, Reddy S: **Increased sickling of parasitised erythrocytes as mechanism of resistance against malaria in the sickle-cell trait.** *Lancet* 1970, **1:**319-321.

7. Pasvol G, Weatherall DJ, Wilson RJ: **Cellular mechanism for the protective effect of haemoglobin S against P. falciparum malaria.** *Nature* 1978, **274:**701-703.

8. Ayi K, Turrini F, Piga A, Arese P: **Enhanced phagocytosis of ring-parasitized mutant erythrocytes: a common mechanism that may explain protection against falciparum malaria in sickle trait and beta-thalassemia trait.** *Blood* 2004, **104:**3364-3371.

9. Bedu-Addo G, Meese S, Mockenhaupt FP: **An ATP2B4 Polymorphism Protects Against Malaria in Pregnancy.** *The Journal of infectious diseases* 2013, **207:**1600-1603.

10. Gazarini ML, Thomas AP, Pozzan T, Garcia CR: **Calcium signaling in a low calcium environment: how the intracellular malaria parasite solves the problem.** *The Journal of cell biology* 2003, **161:**103-110.

11. McMorran BJ, Marshall VM, de Graaf C, Drysdale KE, Shabbar M, Smyth GK, Corbin JE, Alexander WS, Foote SJ: **Platelets kill intraerythrocytic malarial parasites and mediate survival to infection.** *Science (New York, NY)* 2009, **323:**797-800.

12. Bridges DJ, Bunn J, van Mourik JA, Grau G, Preston RJ, Molyneux M, Combes V, O'Donnell JS, de Laat B, Craig A: **Rapid activation of endothelial cells enables Plasmodium falciparum adhesion to platelet-decorated von Willebrand factor strings.** *Blood* 2010, **115:**1472-1474.

13. Varga-Szabo D, Braun A, Nieswandt B: **Calcium signaling in platelets.** *Journal of thrombosis and haemostasis : JTH* 2009, **7:**1057-1066.

14. Szewczyk MM, Davis KA, Samson SE, Simpson F, Rangachari PK, Grover AK: **Ca2+-pumps and Na2+-Ca2+-exchangers in coronary artery endothelium versus smooth muscle.** *Journal of cellular and molecular medicine* 2007, **11:**129-138.

15. Lehmann JM, Moore LB, Smith-Oliver TA, Wilkison WO, Willson TM, Kliewer SA: **An antidiabetic thiazolidinedione is a high affinity ligand for peroxisome proliferator-activated receptor gamma (PPAR gamma).** *The Journal of biological chemistry* 1995, **270:**12953-12956.

16. Williams SM, Canter JA, Crawford DC, Moore JH, Ritchie MD, Haines JL: **Problems with genome-wide association studies.** *Science (New York, NY)* 2007, **316:**1840-1842.

17. Saxena R, Voight BF, Lyssenko V, Burtt NP, de Bakker PI, Chen H, Roix JJ, Kathiresan S, Hirschhorn JN, Daly MJ, Hughes TE, Groop L, Altshuler D, Almgren P, Florez JC, Meyer J, Ardlie K, Bengtsson Bostrom K, Isomaa B, Lettre G, Lindblad U, Lyon HN, Melander O, Newton-Cheh C, Nilsson P, Orho-Melander M, Rastam L, Speliotes EK, Taskinen MR, Tuomi T*, et al*: **Genome-wide association analysis identifies loci for type 2 diabetes and triglyceride levels.** *Science (New York, NY)* 2007, **316:**1331-1336.

18. Scott LJ, Mohlke KL, Bonnycastle LL, Willer CJ, Li Y, Duren WL, Erdos MR, Stringham HM, Chines PS, Jackson AU, Prokunina-Olsson L, Ding CJ, Swift AJ, Narisu N, Hu T, Pruim R, Xiao R, Li XY, Conneely KN, Riebow NL, Sprau AG, Tong M, White PP, Hetrick KN, Barnhart MW, Bark CW, Goldstein JL, Watkins L, Xiang F, Saramies J*, et al*: **A genome-wide association study of type 2 diabetes in Finns detects multiple susceptibility variants.** *Science (New York, NY)* 2007, **316:**1341-1345.

19. Zeggini E, Weedon MN, Lindgren CM, Frayling TM, Elliott KS, Lango H, Timpson NJ, Perry JR, Rayner NW, Freathy RM, Barrett JC, Shields B, Morris AP, Ellard S, Groves CJ, Harries LW, Marchini JL, Owen KR, Knight B, Cardon LR, Walker M, Hitman GA, Morris AD, Doney AS, McCarthy MI, Hattersley AT: **Replication of genome-wide association signals in UK samples reveals risk loci for type 2 diabetes.** *Science (New York, NY)* 2007, **316:**1336-1341.

20. Choi JH, Banks AS, Kamenecka TM, Busby SA, Chalmers MJ, Kumar N, Kuruvilla DS, Shin Y, He Y, Bruning JB, Marciano DP, Cameron MD, Laznik D, Jurczak MJ, Schurer SC, Vidovic D, Shulman GI, Spiegelman BM, Griffin PR: **Antidiabetic actions of a non-agonist PPARgamma ligand blocking Cdk5-mediated phosphorylation.** *Nature* 2011, **477:**477-481.

21. Rosenson RS, Wright RS, Farkouh M, Plutzky J: **Modulating peroxisome proliferator-activated receptors for therapeutic benefit? Biology, clinical experience, and future prospects.** *American heart journal* 2012, **164:**672-680.
